# Protein-Based Nanoparticle Vaccines for SARS-CoV-2

**DOI:** 10.3390/ijms222413445

**Published:** 2021-12-14

**Authors:** Hyo-Dong Sung, Nayeon Kim, Yeram Lee, Eun Jung Lee

**Affiliations:** Department of Chemical Engineering, School of Applied Chemical Engineering, Kyungpook National University, Daegu 41566, Korea; gyehd0314@knu.ac.kr (H.-D.S.); nykim0310@knu.ac.kr (N.K.); yeram0301@knu.ac.kr (Y.L.)

**Keywords:** SARS-CoV-2, protein nanoparticles, vaccines, nanovaccine, nanomedicine, protein-based nanotechnology

## Abstract

The pandemic caused by the severe acute respiratory syndrome coronavirus-2 (SARS-CoV-2) has upended healthcare systems and economies around the world. Rapid understanding of the structural biology and pathogenesis of SARS-CoV-2 has allowed the development of emergency use or FDA-approved vaccines and various candidate vaccines. Among the recently developed SARS-CoV-2 candidate vaccines, natural protein-based nanoparticles well suited for multivalent antigen presentation and enhanced immune stimulation to elicit potent humoral and cellular immune responses are currently being investigated. This mini-review presents recent innovations in protein-based nanoparticle vaccines against SARS-CoV-2. The design and strategy of displaying antigenic domains, including spike protein, receptor-binding domain (RBD), and other domains on the surface of various protein-based nanoparticles and the performance of the developed nanoparticle-based vaccines are highlighted. In the final part of this review, we summarize and discuss recent advances in clinical trials and provide an outlook on protein-based nanoparticle vaccines.

## 1. Introduction

Coronavirus disease 2019 (COVID-19) is an infectious disease caused by severe acute respiratory syndrome coronavirus-2 (SARS-CoV-2), which first appeared in Wuhan, People’s Republic of China, in late 2019 and has since spread around the world, resulting in a pandemic [1,2]. The SARS-CoV-2 pandemic has disrupted global public health and economies in 216 countries, with over 49 million deaths since 29 December, 2019 [3].

Among the recently developed vaccine candidates for SARS-CoV-2, natural protein-based nanoparticles that can confer stronger and broader protective immunity have been shown to be effective [4,5,6,7,8,9,10,11,12,13,14]. A recent phase 3 clinical trial has demonstrated the Novavax vaccine, a recombinant nanoparticle vaccine made of a stabilized form of the coronavirus spike (S) protein, to be safe with an efficacy of 89.7% [15,16]. Additionally, ferritin-based protein nanoparticles presenting the SARS-CoV-2 S protein are entering phase 1 clinical trials (NCT04784767). These nanoparticle-based vaccine technologies can enhance the immunogenicity and stability of soluble antigens by multivalent antigen display on their surface [17,18,19,20,21,22,23]. They can facilitate numerous immunological processes, including the efficient delivery of antigens to lymph nodes, retention of follicular dendritic and helper T cells, and generation, activation, and expansion of B cells, including memory B cells and long-lived plasma cells [18,24,25,26,27,28,29,30,31,32,33,34]. In particular, protein-based nanoparticles have no special biosafety environmental issues in their manufacturing procedures and potentially greater accessibility to the public with reduced production costs [12,35,36,37,38,39]. Thus, protein-based nanoparticles have been widely used as a delivery platform for various vaccines and drugs. A more detailed description of the biological function, structure, and geometry of various protein nanoparticles and their application in nanomedicine, including the development of other vaccines, are described in excellent recent reviews [40,41,42,43,44,45,46].

**Figure 1:**
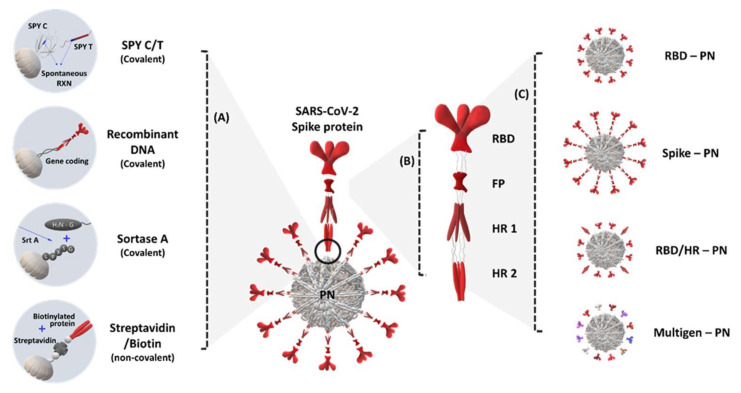
Application of protein nanoparticles (PN) that elicit severe acute respiratory syndrome coronavirus-2 (SARS-CoV-2)- and SARS variant-specific immune response: (**A**) methods for ligating PN components, nanoparticles, and immunogens based on covalent (SpyCatcher/SpyTag, recombinant DNA, and sortase-mediated bonding) and non-covalent (streptavidin/biotin) interactions; (**B**) schematic structure of the SARS-CoV-2 spike (S) protein; (**C**) type of immunogen applications for displaying PNs from the partial epitope region of SARS-CoV-2 to other types of receptor-binding domains (RBDs) and S proteins; SPY C/T, SpyCatcher/SpyTag; HR, heptad repeat.

This review mainly focuses on recent innovations in protein-based nanoparticle vaccines for protection against SARS-CoV-2 (Figure 1). First, the design and strategy of displaying antigenic domains, including S protein, receptor-binding domain (RBD), and other domains, into various protein nanoparticles are discussed. The performance of these engineered protein nanoparticle vaccines for protection against SARS-CoV-2 in mice, human angiotensin-converting enzyme 2 (ACE2) transgenic mice, rabbits, hamsters, ferrets, and macaques, and their ability to protect against other viruses in the Coronaviridae family is highlighted. Finally, we present a summary and perspective of recent advances in protein nanoparticle vaccines involving clinical trials.

## 2. S Protein Domain-Conjugated (Presenting) Protein Nanoparticles

SARS-CoV-2 is a positive-strand genomic RNA virus, and its RNA encodes non-structural proteins such as proteases and RNA polymerase and major structural proteins, including nucleocapsid, membrane, envelope, and S protein [47]. SARS-CoV-2 has a similar cell entry mechanism to other coronaviruses. The S protein protruding from the viral surface mediates host cell receptor recognition, viral attachment, and entry into the host cell via the formation of a trimeric hairpin structure [7]. The S protein mainly consists of S1 and S2 domains. First, the S1 domain binds to the ACE2 receptor on human cell surfaces through its RBD. Then, the S1 domain is cleaved by the host cellular protease furin, followed by the mediation of fusion of the viral envelope and host cell membrane by the S2 domain through its heptad repeat 1 (HR1), heptad repeat 2 (HR2), and fusion peptide domain. Therefore, the S protein is an ideal target for vaccine development due to its biological functions, and most of the currently developed vaccines against SARS-CoV-2 mainly target the S protein as an immunogen.

Powell et al. first reported S protein-conjugated protein nanoparticles based on *Helicobacter pylori* ferritin (Table 1) [48]. *H. pylori* ferritin self-assembles into 24 multimeric spherical nanoparticles approximately 12 nm in size [49]. Primarily, three-fold symmetric axes of the ferritin N-terminus have been used to display trimeric complexes, including surface glycoprotein antigens such as human immunodeficiency virus-1 (HIV-1) and the Epstein–Barr virus [12,36]. Using a similar approach, the full-length S protein or a C-terminal 70 amino acid-deleted S protein was genetically fused to the N-terminus of *H. pylori* ferritin and successfully produced in human Expi293 cells. Cryogenic electron microscopy (cryo-EM) images showed S protein-displaying nanoparticles, and two-dimensional class averages and single-particle analysis confirmed that the octahedral nanoparticles had eight trimeric S antigens on their surfaces. The S protein-displaying nanoparticles with Quil-A/monophosphoryl lipid A successfully elicited a more consistent neutralizing antibody response compared with that with a trimeric form of S protein using the trimeric coiled-coil protein GCN4 and higher S-pseudotyped viral neutralizing titers than those in convalescent COVID-19 patient plasma, even in a single dose.

Icosahedral protein nanoparticles with 120 subunits have been computationally designed and developed as vaccines against HIV-1, the respiratory syncytial virus, and influenza via two-component self-assembly, such as I53-50 and dn5 [10,62,63]. Brouwer et al. developed a SARS-CoV-2 vaccine based on I53-50 nanoparticles composed of 20 trimeric (I53.50A.1NT1) and 12 pentameric (I53.50B.4PT1) subunits [55]. I53.50A.1NT1 was genetically conjugated with the S protein, and the recombinant fusion protein was purified as a trimeric complex using size exclusion chromatography. Then, the trimeric SARS-CoV-2 S-I53.50A.1NT1 was incubated with pentameric I53.50B.4PT1, resulting in S protein-displaying nanoparticles with a diameter of ~30 nm. The multivalent display of the S protein of I53-50 nanoparticles enhances cognate B cell activation in vitro compared with a trimeric form of the S protein (SARS-CoV-2 S-I53.50A.1NT1). The S protein-displaying I53-50 nanoparticles successfully induced neutralizing antibody responses in mice and rabbit models and S protein-specific B and T cell responses in cynomolgus macaques. Furthermore, these nanoparticles showed potent protective efficacy against 10- to 100-fold higher doses of SARS-CoV-2 challenge compared with other studies with reduced viral subgenomic RNA replication in tracheal and nasopharyngeal swabs.

As SARS-CoV-2 vaccine candidates, other types of protein nanoparticles such as the coat protein of the RNA bacteriophage MS2, dihydrolipoyl acetyltransferase (E2p) from *Bacillus* *stearothermophilus,* and computationally designed I3-01v9 have been utilized to display the S protein [45,47]. MS2 consists of 90 homodimers, resulting in 180-subunit icosahedral nanoparticles with a diameter of ~30 nm. Biotin-fused S protein was added to the surface of streptavidin-MS2 nanoparticles using streptavidin and biotin-specific binding systems [58]. Specifically, in addition to a prefusion-stabilized S protein (S2Pro), which contains two proline substitutions and is mostly used in other studies, Hexapro (S6Pro), a variant of S2Pro that contains six proline substitutions and higher stability and expression yield than that of S2Pro, was used in this study. A single immunization of S2Pro- or S6Pro-displaying MS2 with Alhydrogel (AH) effectively protected Syrian hamsters from SARS-CoV-2 infection with rapid elimination of the virus in the nasal turbinates and viral titers more than 150- (S2Pro-MS2) and 700-fold (S6Pro-MS2) lower than those in controls.

Zhu et al. developed various SARS-CoV-2 nanoparticle vaccines using 24-mer ferritin, 60-mer E2p, and 60-mer I3-01v9 [60]. They designed a stable S protein (S2GΔHR2) with a deleted HR-2 stalk and two substituted glycine residues, and genetically conjugated S2GΔHR2 with ferritin, E2p, and I3-01v9. Additionally, S2GΔHR2-E2p and -I3-01v9 had genetically fused locking domains and PADRE, a 13-amino acid pan-DR epitope that activated CD4^+^ T cells, in their inner cavity. These nanoparticles produced higher neutralizing antibody responses in mouse models than did soluble full-length SP2 and S2GΔHR2, with their average EC50 titers corresponding to their size (ferritin < E2p < I3-01v9).

A recently developed nanoparticle vaccine called S protein ferritin nanoparticle (SpFN) developed by the Walter Reed Army Institute of Research of the US Army Medical Research and Development Command (USAMRDC) is being actively investigated in phase 1 trials [13,50,51]. SpFN was developed by the genetic fusion of prefusion-stabilized S protein and *H. pylori* ferritin. First, the efficacy of SpFN was evaluated in a mouse model with two different adjuvants, AH and army liposome formulation containing QS-21 (ALFQ) [50]. SpFN with ALFQ showed significantly higher recruitment and activation of classical and non-classical antigen-presenting cells and a T helper 1 cell (Th1)-based cellular response than SpFN with AH. Moreover, this combination induced a SARS-CoV2 spike epitope (VNFNFNGL; aa 539–546)-specific polyfunctional CD8+ T cell response and killed the peptide-pulsed target cells. This epitope is also present in SARS-CoV1, possibly suggesting the generation of cross-reactive T cells. The vaccination efficacy of SpFN with ALFQ was further evaluated for potent neutralizing antibody responses and neutralizing activity against live virus, pseudovirus, and SARS-CoV-2 protection in rhesus macaques [51]. In this study, SpFN with ALFQ showed a strong cellular immune response, including a Th1-based S protein-specific CD4^+^ T cell response and reduced viral titer upon high-dose SARS-CoV-2 infection in the lung parenchyma and the upper and lower airways. Neutralizing activity against authentic and pseudo-SARS-CoV-2 variants of concern (VOC) and SARS-CoV-1 was induced by vaccination with SpFN. Therefore, the generation of cross-reactive T cells by SpFN with ALFQ may provide protection against other coronavirus strains and SARS-CoV-2 VOCs.

## 3. RBD and Other Domain-Conjugated (Presenting) Protein Nanoparticles

A high-resolution cryo-EM study of the SARS-CoV-2 structure and interface mutation scanning revealed that the RBD in the S protein of SARS-CoV-2, the key binding interface, recognizes the ACE2 receptor in host cells [64,65]. Therefore, the RBD domain is being studied as a promising target for designing candidate SARS-CoV-2 vaccines.

Walls et al. first reported a SARS-CoV-2 nanoparticle vaccine using the RBD domain [56]. I53-50, a two-component icosahedral protein nanoparticle, was used to display the RBD domain. Trimeric I53-50A was genetically fused to the RBD domain with 8, 12, or 16 glycine and serine flexible linkers to present the native trimeric form of RBD and mixed with pentameric I53-50B, resulting in a SARS-CoV-2 nanoparticle vaccine displaying 60 copies of RBD on its surface. This nanoparticle vaccine showed an enhanced binding profile against human ACE2 (hACE2) and physical and antigenic stability compared with the monomeric form of RBD and the trimeric form of S2Pro. In BALB/c mice and mice with human immune repertoire (Kymab Darwin mice), strong neutralizing antibody responses can be induced by I53-50-based nanoparticle vaccines. These nanoparticle vaccines present potent neutralizing activity against pseudo and live SARS-CoV-2, whereas the monomeric form of RBD and S2Pro showed little to no neutralizing effect. In particular, although linker length and antigenic valency do not substantially affect the overall immunogenicity, RBD I53-50 nanoparticles with 12 and 16 glycine and serine linkers induced 10-fold higher neutralizing antibody titers and neutralizing activity compared with S2Pro. Furthermore, RBD-specific germinal center B cells, which are essential for forming a durable B cell memory, were significantly increased with RBD I53-50 nanoparticle treatment compared with those with treatment with the monomeric forms of RBD and S2Pro.

Various types of protein nanoparticles of different sizes and antigen valencies are being compared as potential SARS-CoV-2 vaccine platforms [20,60,61]. Zeng et al. utilized 24-subunit *H. pylori* ferritin, computationally designed and optimized 60-subunit mi3 and 120-subunit I53-50, and developed three different RBD-displaying nanoparticle vaccines using a spy catcher (SpyCatcher) and tag (SpyTag) system [20]. The bacteria-derived SpyCatcher and SpyTag pair efficiently formed covalent bonds by simple mixing. Therefore, the ligation strategy based on SpyCatcher-SpyTag has been utilized to display HIV, hepatitis B virus, and SARS-CoV-2 antigens on the nanoparticle scaffold [30,66]. The produced RBD-SpyTag in HEK293F cells was incubated with the produced SpyCatcher nanoparticles in *Escherichia coli*, resulting in RBD-displaying nanoparticles. The three RBD-displaying nanoparticles (RBD-ferritin, RBD-mi3, and RBD-I53-50) showed high thermal stability, which may benefit commercial production and supply. Additionally, they presented significantly higher binding ability against hACE2 and RBD-specific neutralizing antibody (CB6) and neutralizing antibody titers in mouse models than monomeric RBD. In particular, RBD-mi3 and RBD-I53-50 elicited a higher neutralization effect than RBD-ferritin, indicating that nanoparticle vaccines with higher antigen valency could produce more effective immune responses.

Other nano-scaffolds, including bann (β-annulus-scaffold peptide from the tomato bushy stunt virus), bullfrog-*H. pylori* hybrid ferritin, AaLS (lumazine synthase from *Aquifex aeolicus*), and foldon from T4 bacteriophage fibiritin have been investigated and compared as potential RBD-nano-scaffold plasmid DNA vaccines [61]. As a result, these nano-scaffold vaccines showed potent neutralizing antibody responses compared with RBD alone. However, in this study, given that foldon with six copies of RBD elicits stronger neutralization effects than AaLS with 60 copies of RBD, nano-scaffold sizes and antigen valency on the surface are not the only main factors contributing to vaccine efficacy. Therefore, bann, a small scaffolding domain with many antigen valencies, might be an ideal vaccine platform because of its strongly augmented immune response against the antigen and minimal antibody response against the nano-scaffold itself compared with those with large scaffold domains.

Currently, owing to the risk of emerging variants of SARS-CoV-2 and other zoonotic viruses, research into vaccine development and cross-reaction efficacy analysis against other viruses is being expedited [35,52,57,67]. Haynes et al. developed RBD-displaying ferritin using the sortase A reaction [52]. RBD with a sortase A donor sequence was conjugated to *H. pylori* ferritin with a sortase A acceptor sequence, resulting in 24 RBD-displaying ferritin nanoparticle vaccines. The nanoparticle vaccine successfully induced neutralizing SARS-CoV-2 RBD-specific antibody responses and protection against SARS-CoV-2 in a macaque model. In particular, cross-neutralizing antibody responses against SARS-CoV-2 variants B.1.1.7, P.1, and B.1.351., bat CoV, and SARS-CoV-1 were elicited in macaques with RBD-ferritin immunization. Similarly, Halfmann et al. designed RBD-conjugated mi3 nanoparticles using SpyCatcher-SpyTag systems and tested their efficacy against various viruses [67]. The nanovaccine induced potent cross-reactive antibodies against SARS-CoV-2, SARS-CoV-1, and SARS-CoV-2 variants B.1.1.7, P.1, and B.1.351. Moreover, neutralizing antibody responses against the above five viruses and important VOC Delta variants (B.1.617.2) were demonstrated.

In another report, given that 97.9% of patients who recovered from SARS-CoV-2 showed a high IgG-specific antibody titer against the HR domain, ferritin nanoparticle vaccines have been developed with additional HR-labeled surfaces along with RBD [35]. Based on sequence homology analyses, HR1 and HR2 are more highly conserved than RBD against three SARS-CoV-2 strains (Wuhan-HU-1, SYSU-IHV, and USA-IA-6399) and six human pathogenic coronaviruses (SARS-CoV Tor2, Middle East respiratory syndrome-related coronavirus [MERS-CoV] EMC, human coronavirus [hCoV]-HKU1, hCoV-OC43, hCoV-NL63, and hCoV-229E), five bat coronaviruses, and two pangolin coronaviruses. Both RBD- and HR-displaying nanoparticle vaccines elicited cross-reactive neutralizing antibody responses against SARS-CoV, MERS-CoV, HCoV-229E, HCoVOC43, and RATG13.

Cohen et al. designed heterotypic mosaic nanoparticle vaccines by co-displaying SARS-CoV-2 RBD with RBDs from other animal coronaviruses to evaluate whether the heterotypic nanoparticles can elicit cross-reactive antibody responses. They displayed four or eight of the 12 RBDs on the surface of mosaic nanoparticles using SpyCatcher-SpyTag ligation. Heterotypic nanoparticle vaccines successfully induced broader anti-coronavirus responses than homotypic nanoparticle vaccines displaying SARS-CoV-2 RBD alone, suggesting that the co-display strategy is advantageous for inducing cross-immunity against zoonotic sarbecoviruses.

## 4. Conclusions and Perspectives

Viral vector vaccines from Astra Zeneca and Johnson & Johnson, mRNA-based vaccines from Pfizer and Moderna, and inactivated vaccines from Sinovac Biotech have been distributed worldwide, and hundreds of SARS-CoV-2 vaccine candidates are currently being evaluated in clinical trials. Among them, recombinant protein subunit vaccines using S protein or RBD are an attractive alternative to inactivated, viral vector, and mRNA-based vaccines owing to their track records of safety. However, despite extensive efforts to develop and apply the S protein or RBD-based subunit as vaccine candidates, their low immunogenicity remains a hindrance.

Compared with the monomeric form of recombinant S protein subunit vaccines, nanoparticle-based vaccines provide multivalent S protein or RBD display. This repetitive array promotes various immunological events, including robust B cell activation, memory B cell expansion, and retention of follicular dendritic cells. Therefore, protein nanoparticle-based vaccines have proven to have enhanced efficacy, neutralizing antibody responses, and specific humoral and cellular immune responses at lower doses than the S protein subunit vaccine. Additionally, inherent stability, the lack of a strict cold-chain supply, and no special biosafety environment concern in manufacturing procedures with reduced production costs are major advantages of protein nanoparticle-based SARS-CoV-2 vaccines [38]. Therefore, nanoparticle vaccines against SARS-CoV-2 (IVX-411) and other viruses (malaria (NCT04296279), Epstein–Barr virus (NCT04645147), and influenza (NCT03186781; NCT03814720; NCT04579250)) are currently being investigated in clinical trials.

On the other hand, the protein nanoparticle-based vaccine platform generates strong cross-reactive immunity against emerging SARS-CoV-2 VOCs and other zoonotic coronaviruses such as SARS-CoV and MERS-CoV. Despite mRNA-based vaccines from Pfizer and Moderna and viral vector vaccines from Astra Zeneca and Johnson & Johnson showing strong protective efficacy against the original Wuhan-Hu-1 strain (wild-type) and slowing the infection rate, the recent emergence of rapidly evolving SARS-CoV-2 VOCs, including Delta and Mu coronavirus variants, presents new challenges. Additionally, two other zoonotic betacoronaviruses, SARS-CoV and MERS-CoV, have appeared in human populations over the past 20 years, and the possibility of future potential zoonotic coronavirus emergence exists, making the next pandemic imminent. From this point of view, protein nanoparticle-based vaccines allow the development of a next-generation vaccine platform for protecting against SARS-CoV-2 VOCs and other zoonotic coronaviruses. They could provide strong protection against the destructive effects of pandemics on the public health system and economy.

## Figures and Tables

**Table 1:** Summary of advances in protein nanoparticle vaccines for severe acute respiratory syndrome coronavirus-2 (SARS-CoV-2).

Nanoparticle	Immunogen	In Vivo Model	Adjuvant	Route	Dose (μg)	Infection	Ref.
Ferritin (Bullfrog and *Helicobacter pylori*)	S protein RBD protein RBD protein and N-terminal domain	BALB/c mouse C57BL/6 mouse K18-hACE2 mouse	ALFQ or Alhydrogel	Intramuscular	0.08–10	Intranasal (1.25 × 10^4^ PFU)	[13]
	S protein	C57BL/6 mouse	ALFQ or Alhydrogel	Intramuscular	10	N/A	[50]
	S protein	Rhesus macaque	ALFQ	Intramuscular	5–50	Intranasal and Intratracheal (1.00 × 10^6^ TCID50)	[51]
	RBD protein	Ferret	AddaVax	Intramuscular	15	Intranasal (1.00 × 10^5^–1.00 × 10^6^ TCID50)	[7]
				Intramuscular and Intranasal			
Ferritin (*Helicobacter pylori*)	RBD protein	Cynomol gusmacaque	3M-052 and Alhydrogel	Intramuscular	100	Intranasal and Intratracheal (1.00 × 10^5^ PFU)	[52]
		Rhesus macaque					
	RBD protein and HR domain	BALB/c mouse	Sigma Adjuvant System	Subcutaneous	10	Intranasal (4.00 × 10^4^ FFU)	[35]
		C57BL/6 mouse(hACE2 transgenic)					
		Rhesus macaque		Intramuscular	50		
	S protein	C57BL/6 mouse	Sigma Adjuvant System	Subcutaneous	20	N/A	[53]
		Syrian golden hamster		Intramuscular	100	Intranasal (1.99 × 10^4^ TCID50)	
	S protein	BALB/c mouse	Quil-A and MPLA	Subcutaneous	0.1–20	N/A	[48]
Ferritin *(Pyrococcus furiosus)*	RBD protein	C57BL/6 mouse	CpG 1826	Subcutaneous	12.3–30.7	N/A	[54]
I53-50 (Artificial)	S protein	BALB/c mouse	Poly(I:C)	Subcutaneous	13	N/A	[55]
		New Zealand white rabbit	Squalene emulsion	Intramuscular	39		
		Cynomolgus macaque	MPLA liposome	Intramuscular	50	Intranasal and Intratracheal (1.00 × 10^6^ PFU)	
	RBD protein	BALB/c mouse	AddaVax	Intramuscular	0.9–5	Intranasal (1.00 × 10^5^ PFU)	[56]
		Kymab Darwin mouse				N/A	
Mi3 (Artificial)	RBD protein (4a, 4b, or 8)	BALB/c mouse	AddaVax	Intraperitoneal	5	N/A	[57]
MS2 (*Emesvirus zinderi*)	S protein	Syrian golden hamster	Alhydrogel	Subcutaneous	60	Intranasal (1.00 × 10^3^ PFU)	[58]
Dps (*Sulfolobus islandicus*)	RBD protein	C57BL/6J mouse	CpG 1668	Subcutaneous	25–50	N/A	[59]
		K18 mouse (hACE2 transgenic)			25	Intranasal (1.00 × 10^4^ PFU)	
	S protein	C57BL/6J mouse			25–50	N/A	
	Nucleocapsid protein	C57BL/6J mouse			25–50	N/A	
I3-01v9 (Artificial)	S protein	BALB/c mouse	AddaVax or Adju-Phos	Intraperitoneal	50	N/A	[60]
E2p (*Geobacillus* *sterothermophilus*)	S protein						
Ferritin (*Helicobacter pylori*)	S protein						
	RBD protein						
Bann (Tomato bushystunt virus)	RBD protein	BALB/c mouse	N/A	Intramuscular, Intranasal, or Sublingual	20 (Plasmid)	Intranasal (70 μL of VSV-S pseudovirus)	[61]
			AddaVax	Intramuscular	100 (protein)		
Foldon (T4 bacteriophage fibritin)			N/A	Intramuscular	20 (Plasmid)		
Ferritin (Bullfrog and *Helicobacter pylori)*			N/A	Intramuscular	20 (Plasmid)		
AaLS (*Aquifex aeolicus*)			N/A	Intramuscular	20 (Plasmid)		
I53-50 (Artificial)	RBD protein	BALB/c mouse	AddaVax or Sigma Adjuvant System	Subcutaneous	11.91	N/A	[20]
Mi3 (Artificial)					9.51		
Ferritin (*Helicobacter pylori*)					9.34		

Abbreviations: ALFQ, army liposome formulation containing QS-21; COVID-19, coronavirus disease 2019; PFU, plaque-forming units; RBD, receptor-binding domain; Th1, T helper 1 cells; APC, antigen-presenting cells; TCID50, tissue culture infectious dose 50; HR, heptad repeat; hACE2, human angiotensin-converting enzyme 2; MPLA, monophosphoryl lipid A; sgRNA, subgenomic RNA; 4a, (SARS-CoV-2, SHC014, RaTG13, Rs4081); 4b, (pang17, RmYN02, Rf1, WIV1); 8, (4a and 4b).

## References

[1] WHO Director-General’s Opening Remarks at the Media Briefing on COVID-19—7 September 2020. https://www.who.int/director-general/speeches/detail/who-director-general-s-opening-remarks-at-the-media-briefing-on-covid-19---7-september-2020.

[2] Coronavirus Resource Center COVID-19 Dashboard by the Center for Systems Science and Engineering (CSSE) at Johns Hopkins University (JHU). https://coronavirus.jhu.edu/map.html.

[3] WHO Coronavirus (COVID-19) Dashboard. https://covid19.who.int/.

[4] Wrapp D., Wang N., Corbett Kizzmekia S., Goldsmith Jory A., Hsieh C.-L., Abiona O., Graham Barney S., McLellan Jason S. (2020). Cryo-EM structure of the 2019-nCoV spike in the prefusion conformation. Science.

[5] Shang J., Ye G., Shi K., Wan Y., Luo C., Aihara H., Geng Q., Auerbach A., Li F. (2020). Structural basis of receptor recognition by SARS-CoV-2. Nature.

[6] Wang Q., Zhang Y., Wu L., Niu S., Song C., Zhang Z., Lu G., Qiao C., Hu Y., Yuen K.-Y. (2020). Structural and Functional Basis of SARS-CoV-2 Entry by Using Human ACE2. Cell.

[7] Kim Y.-I., Kim D., Yu K.-M., Seo H.D., Lee S.-A., Casel M.A.B., Jang S.-G., Kim S., Jung W., Lai C.-J. (2021). Development of Spike Receptor-Binding Domain Nanoparticles as a Vaccine Candidate against SARS-CoV-2 Infection in Ferrets. mBio.

[8] Ingale J., Stano A., Guenaga J., Sharma S.K., Nemazee D., Zwick M.B., Wyatt R.T. (2016). High-Density Array of Well-Ordered HIV-1 Spikes on Synthetic Liposomal Nanoparticles Efficiently Activate B Cells. Cell Rep..

[9] Thompson E.A., Ols S., Miura K., Rausch K., Narum D.L., Spångberg M., Juraska M., Wille-Reece U., Weiner A., Howard R.F. (2018). TLR-adjuvanted nanoparticle vaccines differentially influence the quality and longevity of responses to malaria antigen Pfs25. JCI Insight.

[10] Marcandalli J., Fiala B., Ols S., Perotti M., de van der Schueren W., Snijder J., Hodge E., Benhaim M., Ravichandran R., Carter L. (2019). Induction of Potent Neutralizing Antibody Responses by a Designed Protein Nanoparticle Vaccine for Respiratory Syncytial Virus. Cell.

[11] Yu F., Wang J., Dou J., Yang H., He X., Xu W., Zhang Y., Hu K., Gu N. (2012). Nanoparticle-based adjuvant for enhanced protective efficacy of DNA vaccine Ag85A-ESAT-6-IL-21 against Mycobacterium tuberculosis infection. Nanomedicine.

[12] Kanekiyo M., Wei C.-J., Yassine H.M., McTamney P.M., Boyington J.C., Whittle J.R.R., Rao S.S., Kong W.-P., Wang L., Nabel G.J. (2013). Self-assembling influenza nanoparticle vaccines elicit broadly neutralizing H1N1 antibodies. Nature.

[13] Joyce M.G., Chen W.-H., Sankhala R.S., Hajduczki A., Thomas P.V., Choe M., Chang W., Peterson C.E., Martinez E., Morrison E.B. (2021). SARS-CoV-2 ferritin nanoparticle vaccines elicit broad SARS coronavirus immunogenicity. bioRxiv.

[14] Pati R., Shevtsov M., Sonawane A. (2018). Nanoparticle Vaccines Against Infectious Diseases. Front. Immunol..

[15] Bangaru S., Ozorowski G., Turner H.L., Antanasijevic A., Huang D., Wang X., Torres J.L., Diedrich J.K., Tian J.H., Portnoff A.D. (2020). Structural analysis of full-length SARS-CoV-2 spike protein from an advanced vaccine candidate. Science.

[16] Heath P.T., Galiza E.P., Baxter D.N., Boffito M., Browne D., Burns F., Chadwick D.R., Clark R., Cosgrove C., Galloway J. (2021). Safety and Efficacy of NVX-CoV2373 Covid-19 Vaccine. N. Engl. J. Med..

[17] Gause K.T., Wheatley A.K., Cui J., Yan Y., Kent S.J., Caruso F. (2017). Immunological Principles Guiding the Rational Design of Particles for Vaccine Delivery. ACS Nano.

[18] Link A., Zabel F., Schnetzler Y., Titz A., Brombacher F., Bachmann M.F. (2012). Innate immunity mediates follicular transport of particulate but not soluble protein antigen. J. Immunol..

[19] Wang N., Shang J., Jiang S., Du L. (2020). Subunit Vaccines against Emerging Pathogenic Human Coronaviruses. Front. Microbio..

[20] Kang Y.-F., Sun C., Zhuang Z., Yuan R.-Y., Zheng Q., Li J.-P., Zhou P.-P., Chen X.-C., Liu Z., Zhang X. (2021). Rapid Development of SARS-CoV-2 Spike Protein Receptor-Binding Domain Self-Assembled Nanoparticle Vaccine Candidates. ACS Nano.

[21] Kih M., Lee E.J., Lee N.K., Kim Y.K., Lee K.E., Jeong C., Yang Y., Kim D.-H., Kim I.-S. (2018). Designed trimer-mimetic TNF superfamily ligands on self-assembling nanocages. Biomaterials.

[22] Je H., Nam G.-H., Kim G.B., Kim W., Kim S.R., Kim I.-S., Lee E.J. (2021). Overcoming therapeutic efficiency limitations against TRAIL-resistant tumors using re-sensitizing agent-loaded trimeric TRAIL-presenting nanocages. J. Control. Release.

[23] Choi Y., Nam G.-H., Kim G.B., Kim S., Kim Y.K., Kim S.A., Kim H.-J., Lee E.J., Kim I.-S. (2021). Nanocages displaying SIRP gamma clusters combined with prophagocytic stimulus of phagocytes potentiate anti-tumor immunity. Cancer Gene Ther..

[24] Hu X., Deng Y., Chen X., Zhou Y., Zhang H., Wu H., Yang S., Chen F., Zhou Z., Wang M. (2017). Immune Response of A Novel ATR-AP205-001 Conjugate Anti-hypertensive Vaccine. Sci. Rep..

[25] Pardi N., Hogan M.J., Naradikian M.S., Parkhouse K., Cain D.W., Jones L., Moody M.A., Verkerke H.P., Myles A., Willis E. (2018). Nucleoside-modified mRNA vaccines induce potent T follicular helper and germinal center B cell responses. J. Exp. Med..

[26] López-Sagaseta J., Malito E., Rappuoli R., Bottomley M.J. (2016). Self-assembling protein nanoparticles in the design of vaccines. Comput. Struct. Biotechnol. J..

[27] Slifka M.K., Amanna I.J. (2019). Role of Multivalency and Antigenic Threshold in Generating Protective Antibody Responses. Front. Immunol..

[28] Kelly H.G., Kent S.J., Wheatley A.K. (2019). Immunological basis for enhanced immunity of nanoparticle vaccines. Expert Rev. Vaccines.

[29] Tokatlian T., Read B.J., Jones C.A., Kulp D.W., Menis S., Chang J.Y.H., Steichen J.M., Kumari S., Allen J.D., Dane E.L. (2019). Innate immune recognition of glycans targets HIV nanoparticle immunogens to germinal centers. Science.

[30] Wang W., Zhou X., Bian Y., Wang S., Chai Q., Guo Z., Wang Z., Zhu P., Peng H., Yan X. (2020). Dual-targeting nanoparticle vaccine elicits a therapeutic antibody response against chronic hepatitis B. Nat. Nanotechnol..

[31] Kato Y., Abbott R.K., Freeman B.L., Haupt S., Groschel B., Silva M., Menis S., Irvine D.J., Schief W.R., Crotty S. (2020). Multifaceted Effects of Antigen Valency on B Cell Response Composition and Differentiation In Vivo. Immunity.

[32] Zhang Y.-N., Lazarovits J., Poon W., Ouyang B., Nguyen L.N.M., Kingston B.R., Chan W.C.W. (2019). Nanoparticle Size Influences Antigen Retention and Presentation in Lymph Node Follicles for Humoral Immunity. Nano Lett..

[33] Kim G.B., Sung H.-D., Nam G.-H., Kim W., Kim S., Kang D., Lee E.J., Kim I.-S. (2021). Design of PD-1-decorated nanocages targeting tumor-draining lymph node for promoting T cell activation. J. Control. Release.

[34] Lee E.J., Nam G.H., Lee N.K., Kih M., Koh E., Kim Y.K., Hong Y., Kim S., Park S.Y., Jeong C. (2018). Nanocage-Therapeutics Prevailing Phagocytosis and Immunogenic Cell Death Awakens Immunity against Cancer. Adv. Mater..

[35] Ma X., Zou F., Yu F., Li R., Yuan Y., Zhang Y., Zhang X., Deng J., Chen T., Song Z. (2020). Nanoparticle Vaccines Based on the Receptor Binding Domain (RBD) and Heptad Repeat (HR) of SARS-CoV-2 Elicit Robust Protective Immune Responses. Immunity.

[36] Kanekiyo M., Bu W., Joyce M.G., Meng G., Whittle J.R., Baxa U., Yamamoto T., Narpala S., Todd J.P., Rao S.S. (2015). Rational Design of an Epstein-Barr Virus Vaccine Targeting the Receptor-Binding Site. Cell.

[37] Kelly H.G., Tan H.-X., Juno J.A., Esterbauer R., Ju Y., Jiang W., Wimmer V.C., Duckworth B.C., Groom J.R., Caruso F. (2020). Self-assembling influenza nanoparticle vaccines drive extended germinal center activity and memory B cell maturation. JCI Insight.

[38] He D., Marles-Wright J. (2015). Ferritin family proteins and their use in bionanotechnology. New Biotechnol..

[39] Lee N.K., Lee E.J., Kim S., Nam G.H., Kih M., Hong Y., Jeong C., Yang Y., Byun Y., Kim I.S. (2017). Ferritin nanocage with intrinsically disordered proteins and affibody: A platform for tumor targeting with extended pharmacokinetics. J. Control. Release.

[40] Lv C., Zhang X., Liu Y., Zhang T., Chen H., Zang J., Zheng B., Zhao G. (2021). Redesign of protein nanocages: The way from 0D, 1D, 2D to 3D assembly. Chem. Soc. Rev..

[41] Ren H., Zhu S., Zheng G. (2019). Nanoreactor Design Based on Self-Assembling Protein Nanocages. Int. J. Mol. Sci..

[42] Diaz D., Care A., Sunna A. (2018). Bioengineering Strategies for Protein-Based Nanoparticles. Genes.

[43] Bhaskar S., Lim S. (2017). Engineering protein nanocages as carriers for biomedical applications. NPG Asia Mater..

[44] Lee E.J., Lee N.K., Kim I.-S. (2016). Bioengineered protein-based nanocage for drug delivery. Adv. Drug Del. Rev..

[45] Demchuk A.M., Patel T.R. (2020). The biomedical and bioengineering potential of protein nanocompartments. Biotechnol. Adv..

[46] Nguyen B., Tolia N.H. (2021). Protein-based antigen presentation platforms for nanoparticle vaccines. NPJ Vaccines.

[47] Huang Y., Yang C., Xu X.-F., Xu W., Liu S.-W. (2020). Structural and functional properties of SARS-CoV-2 spike protein: Potential antivirus drug development for COVID-19. Acta Pharmacolol. Sin..

[48] Powell A.E., Zhang K., Sanyal M., Tang S., Weidenbacher P.A., Li S., Pham T.D., Pak J.E., Chiu W., Kim P.S. (2021). A Single Immunization with Spike-Functionalized Ferritin Vaccines Elicits Neutralizing Antibody Responses against SARS-CoV-2 in Mice. ACS Cent. Sci..

[49] Cho K.J., Shin H.J., Lee J.-H., Kim K.-J., Park S.S., Lee Y., Lee C., Park S.S., Kim K.H. (2009). The Crystal Structure of Ferritin from Helicobacter pylori Reveals Unusual Conformational Changes for Iron Uptake. J. Mol. Biol..

[50] Carmen J.M., Shrivastava S., Lu Z., Anderson A., Morrison E.B., Sankhala R.S., Chen W.-H., Chang W.C., Bolton J.S., Matyas G.R. (2021). A spike-ferritin nanoparticle vaccine induces robust innate immune activity and drives polyfunctional SARS-CoV-2-specific T cells. bioRxiv.

[51] Joyce M.G., King H.A.D., Naouar I.E., Ahmed A., Peachman K.K., Cincotta C.M., Subra C., Chen R.E., Thomas P.V., Chen W.-H. (2021). Efficacy of a Broadly Neutralizing SARS-CoV-2 Ferritin Nanoparticle Vaccine in Nonhuman Primates. bioRxiv.

[52] Saunders K.O., Lee E., Parks R., Martinez D.R., Li D., Chen H., Edwards R.J., Gobeil S., Barr M., Mansouri K. (2021). Neutralizing antibody vaccine for pandemic and pre-emergent coronaviruses. Nature.

[53] Gu M., Torres J.L., Greenhouse J., Wallace S., Chiang C.-I., Jackson A.M., Porto M., Kar S., Li Y., Ward A.B. (2021). One dose of COVID-19 nanoparticle vaccine REVC-128 protects against SARS-CoV-2 challenge at two weeks post-immunization. Emerg Microbes Infect..

[54] Wang W., Huang B., Zhu Y., Tan W., Zhu M. (2021). Ferritin nanoparticle-based SARS-CoV-2 RBD vaccine induces a persistent antibody response and long-term memory in mice. Cell. Mol. Immunol..

[55] Brouwer P.J.M., Brinkkemper M., Maisonnasse P., Dereuddre-Bosquet N., Grobben M., Claireaux M., de Gast M., Marlin R., Chesnais V., Diry S. (2021). Two-component spike nanoparticle vaccine protects macaques from SARS-CoV-2 infection. Cell.

[56] Walls A.C., Fiala B., Schäfer A., Wrenn S., Pham M.N., Murphy M., Tse L.V., Shehata L., O’Connor M.A., Chen C. (2020). Elicitation of Potent Neutralizing Antibody Responses by Designed Protein Nanoparticle Vaccines for SARS-CoV-2. Cell.

[57] Cohen A.A., Gnanapragasam P.N.P., Lee Y.E., Hoffman P.R., Ou S., Kakutani L.M., Keeffe J.R., Wu H.-J., Howarth M., West A.P. (2021). Mosaic nanoparticles elicit cross-reactive immune responses to zoonotic coronaviruses in mice. Science.

[58] Chiba S., Frey S.J., Halfmann P.J., Kuroda M., Maemura T., Yang J.E., Wright E.R., Kawaoka Y., Kane R.S. (2021). Multivalent nanoparticle-based vaccines protect hamsters against SARS-CoV-2 after a single immunization. Commun. Bio..

[59] Salzer R., Clark J.J., Vaysburd M., Chang V.T., Albecka-Moreau A., Kiss L., Sharma P., Llamazares A.G., Kipar A., Hiscox J.A. (2021). Single-dose immunisation with a multimerised SARS-CoV-2 receptor binding domain (RBD) induces an enhanced and protective response in mice. FEBS Lett..

[60] He L., Lin X., Wang Y., Abraham C., Sou C., Ngo T., Zhang Y., Wilson I.A., Zhu J. (2021). Single-component, self-assembling, protein nanoparticles presenting the receptor binding domain and stabilized spike as SARS-CoV-2 vaccine candidates. Sci. Adv..

[61] Lainšček D., Fink T., Forstnerič V., Hafner-Bratkovič I., Orehek S., Strmšek Ž., Manček-Keber M., Pečan P., Esih H., Malenšek Š. (2021). A Nanoscaffolded Spike-RBD Vaccine Provides Protection against SARS-CoV-2 with Minimal Anti-Scaffold Response. Vaccines.

[62] Boyoglu-Barnum S., Ellis D., Gillespie R.A., Hutchinson G.B., Park Y.-J., Moin S.M., Acton O., Ravichandran R., Murphy M., Pettie D. (2020). Elicitation of broadly protective immunity to influenza by multivalent hemagglutinin nanoparticle vaccines. bioRxiv.

[63] Brouwer P.J.M., Antanasijevic A., Berndsen Z., Yasmeen A., Fiala B., Bijl T.P.L., Bontjer I., Bale J.B., Sheffler W., Allen J.D. (2019). Enhancing and shaping the immunogenicity of native-like HIV-1 envelope trimers with a two-component protein nanoparticle. Nat. Commun..

[64] Li F., Li W., Farzan M., Harrison S.C. (2005). Structure of SARS coronavirus spike receptor-binding domain complexed with receptor. Science.

[65] Lan J., Ge J., Yu J., Shan S., Zhou H., Fan S., Zhang Q., Shi X., Wang Q., Zhang L. (2020). Structure of the SARS-CoV-2 spike receptor-binding domain bound to the ACE2 receptor. Nature.

[66] Escolano A., Gristick H.B., Abernathy M.E., Merkenschlager J., Gautam R., Oliveira T.Y., Pai J., West A.P., Barnes C.O., Cohen A.A. (2019). Immunization expands B cells specific to HIV-1 V3 glycan in mice and macaques. Nature.

[67] Halfmann P.J., Castro A., Loeffler K., Frey S.J., Chiba S., Kawaoka Y., Kane R.S. (2021). Potent neutralization of SARS-CoV-2 including variants of concern by vaccines presenting the receptor-binding domain multivalently from nanoscaffolds. Bioeng. Transl. Med..

